# The Development of a Multilevel Intervention to Optimise Participant Engagement with an Obesity Prevention Programme Delivered in UK children’s Centres

**DOI:** 10.1007/s11121-021-01205-y

**Published:** 2021-02-01

**Authors:** Wendy Burton, Pinki Sahota, Maureen Twiddy, Julia Brown, Maria Bryant

**Affiliations:** 1 Leeds Institute of Clinical Trials Research, University of Leeds, Leeds, LS2 9JT UK; 2 School of Clinical and Applied Sciences, Leeds Beckett University, City Campus, Leeds, LS1 3HE UK; 3 Institute of Clinical and Applied Health Research, Hull York Medical School, University of Hull, Cottingham Rd, Hull, HU6 7RX UK

**Keywords:** Public health, Intervention, Participant engagement, Obesity prevention, Behaviour Change Wheel, Children’s centres

## Abstract

Poor participant engagement threatens the potential impact and cost-effectiveness of public health programmes preventing meaningful evaluation and wider application. Although barriers and levers to engagement with public health programmes are well documented, there is a lack of proven strategies in the literature addressing these. This paper details the development of a participant engagement intervention aimed at promoting enrolment and attendance to a community-based pre-school obesity prevention programme delivered in UK children’s centres; HENRY (Health, Exercise, Nutrition for the Really Young). *The* Behaviour Change Wheel framework was used to guide the development of the intervention. The findings of a coinciding focused ethnography study identified barriers and levers to engagement with HENRY that informed which behaviours should be targeted within the intervention to promote engagement. A COM-B behavioural analysis was undertaken to identify whether capability, opportunity or motivation would need to be influenced for the target behaviours to occur. APEASE criteria were used to agree on appropriate intervention functions and behaviour change techniques. A multi-level participant engagement intervention was developed to promote adoption of target behaviours that were proposed to promote engagement with HENRY, e.g. ensuring the programme is accurately portrayed when approaching individuals to attend and providing ‘taster’ sessions prior to each programme. At the local authority level, the intervention aimed to increase buy-in with HENRY to increase the level of resource dedicated to engagement efforts. At the centre level, managers were encouraged to widen promotion of the programme and ensure that staff promoted the programme accurately. HENRY facilitators received training to increase engagement during sessions, and parents that had attended HENRY were encouraged to recruit their peers. This paper describes one of the first attempts to develop a theory-based multi-level participant engagement intervention specifically designed to promote recruitment and retention to a community-based obesity prevention programme. Given the challenges to implementing public health programmes with sufficient reach, the process used to develop the intervention serves as an example of how programmes that are already widely commissioned could be optimised to enable greater impact.

## Background

Local authorities in England are responsible for improving the health and well-being of people living in their communities. This includes providing equitable access to public health programmes that promote positive lifestyle behaviours. Populations living in the most deprived areas of England are more likely to have higher rates of smoking, poor mental health and obesity than those (Public Health England 2019) from more affluent areas.

Community-based public health programmes that are adopted and implemented as planned by local authorities have the potential to promote health and reduce heath inequalities. However, a major barrier which hinders their effective implementation is poor participant engagement (enrolment and completion). Poor engagement reduces potential impact of public health programmes, with greater uptake and reach being associated with better outcomes for participants (Bamberger et al. 2014). The cost-effectiveness of programmes is also reduced, with literature showing an increased cost per person when classes do not run with the intended number of people, often resulting in programmes ending prematurely or being cancelled before they start (Lindsay et al. 2014). Further, poor engagement hinders evaluation efforts, preventing wider application.

Engaging participants with public health programmes is known to be a challenge (Morawska et al. 2011). This is particularly pertinent to prevention interventions that are aimed at a general population rather than a targeted group (Spoth and Redmond 2000) in which potential participants may perceive a lack of relevance, experience no clinical symptoms or be hesitant to receive unwanted lifestyle advice (Harte et al. 2018). The literature describes many barriers to engagement with public health interventions such as lack of time, cost of public transport and social and cultural barriers (La Placa and Corlyon 2014) which suggest that programme deliverers should invest resources into the design, delivery and evaluation of engagement strategies aimed at addressing these barriers. Yet studies reporting on such efforts are few, and there is a particular lack of studies that have rigorously evaluated an engagement strategy.

A public health programme that is currently widely delivered in the UK (delivered in 32 local authorities, providing more than 150 programmes per year) is HENRY; Health, Exercise and Nutrition for the Really Young, a pre-school obesity prevention programme predominately delivered in children’s centres. HENRY is an 8-week group parenting programme (2 h per week) that aims to prevent the development of obesity in young children by supporting the whole family to make positive lifestyle change to create a healthy and happy home environment (HENRY 2020). The programme includes elements on parent and child well-being, parenting skills, healthy mealtimes and active lifestyles. Initial evaluation findings of the programme are promising and show that it may have a positive impact on families and practitioners (Willis et al. 2012; Willis et al. 2016). However, implementation data indicate that some local authorities and children’s centres fail to meet their enrolment and engagement targets of eight families per programme and completion of a minimum of five out of the eight sessions, threatening its potential impact and sustainability.

This aim of this paper is to describe the development of a participant engagement intervention aimed at supporting children’s centres and local authorities to promote parent engagement with the existing HENRY programme. Outlined in the paper is the intervention development process which was informed by the Behaviour Change Wheel (Michie et al. 2011) and a description of the final intervention design. Although this intervention is focused on promoting parent engagement with HENRY, it has been developed with transferability in mind so that it has the potential to be adapted for other community-based interventions.

## Methods

### Intervention Development Team

A multi-disciplinary team was convened to develop the participant engagement intervention which included experts in intervention development, obesity, applied health research and behaviour change; a local authority (local government) representative; a HENRY parent; and the chief executive of HENRY. The intervention development team met five times during the 6-month intervention development process (July to December 2015) with tasks completed between meetings. A parent advisory group was also consulted during the intervention development to discuss barriers and facilitators to engagement with HENRY and gain feedback for intervention components.

### Literature Review

Prior to the development of the intervention, a comprehensive review of the relevant literature was conducted to identify interventions that had previously been tried and tested to promote engagement with a public health programme.

### Focused Ethnography Study

During the development of the engagement intervention, a focused ethnography study was undertaken to provide primary evidence about the factors influencing parent engagement with HENRY. Key findings of the ethnography were used to inform the development of the intervention. The ethnography study methods and results have already been published elsewhere (Burton et al. 2019) and are therefore only briefly described here. During the ethnography study, five children’s centres were visited that delivered HENRY across the UK, with 190 h of field observations, 22 staff interviews (commissioners, HENRY co-ordinators, managers and facilitators) and six parent focus groups (36 parents). The aim of the study was to identify barriers and levers to engagement with HENRY within the children’s centre context from the perspective of individuals involved in its implementation along with parents visiting the centre.

### Behaviour Change Wheel Framework

**Fig. 1:**
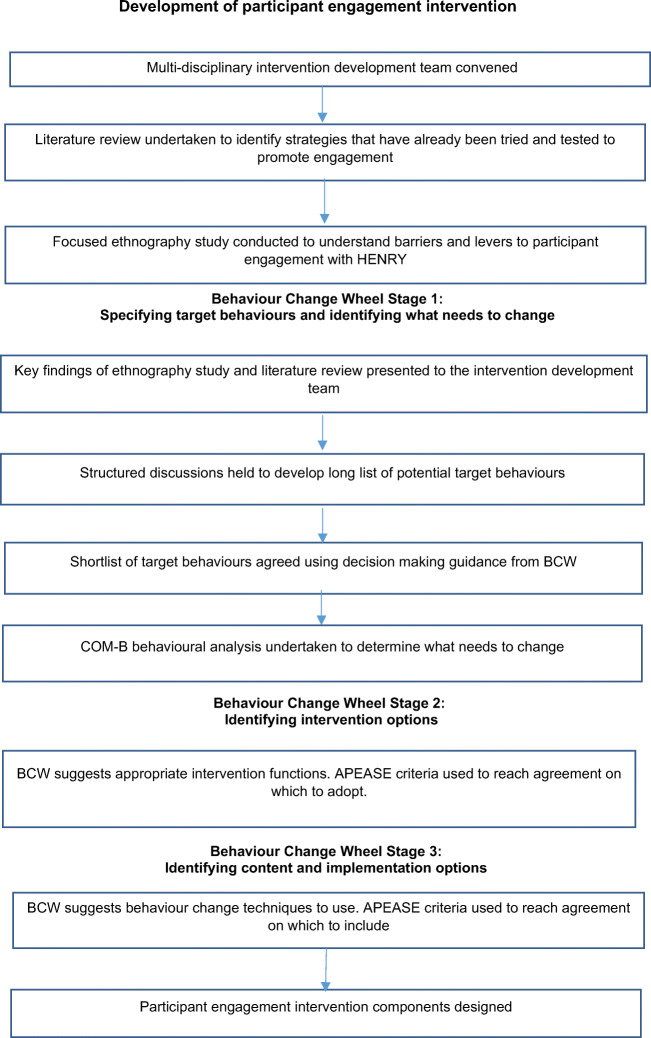
Participant engagement intervention development process

The Behaviour Change Wheel (BCW) (Michie et al. 2011) was used as a guide to develop the intervention which is underpinned by the COM-B (capability, opportunity, motivation) model of behaviour, which proposes that one or more of its behavioural components need to be influenced for behaviour change to occur. The BCW approach involves 3 stages of intervention development: Stage 1, specifying the target behaviours and identifying what needs to change; Stage 2, identifying intervention functions (the ways in which the intervention will operate); and Stage 3, identifying the content and implementation options. The intervention development process we adopted is summarised in Fig. 1.

#### Behaviour Change Wheel Stage 1: Specifying Target Behaviours and Identifying What Needs to Change

##### Defining the Problem in Behavioural Terms

To understand how to promote participant engagement with HENRY, the development team considered data from the ethnography study, key literature surrounding engagement with parenting programmes (e.g. Mytton et al. 2014), the implementation of public health programmes (e.g. Damschroder et al. 2009) and their own experience and expertise to identify the main barriers and levers to engagement. This was translated into a ‘long list’ of target behaviours that could potentially be addressed within the intervention.

##### Selecting Target Behaviours

The BCW advises that the number of behaviours targeted within an intervention should be limited as a small number of successes is more likely to be effective than trying to do too much at once (Michie et al. 2014); therefore, the ‘long list’ of behaviours was narrowed down to a ‘short list’ of target behaviours using decision-making guidance from the BCW. This process involved structured discussions where the team used the evidence to categorise each behaviour as promising, very promising, unpromising but worth considering and unacceptable. Categorisation was achieved by considering the expected impact of the behaviour change, the likelihood of changing the behaviour, anticipated wider impact (‘spill over score’) and the behaviour change measurability. A ranking exercise then took place whereby each development team member individually selected their ‘top ten’ target behaviours from the promising or very promising behaviours list, assigning each a score of 1 to10, considering which were achievable within existing funds and timescales of the delivery period. Team members were permitted to prioritise fewer or more than ten if necessary. The scores were then collated and the highest scoring was added to the short list. Where a team member felt strongly that additional behaviours should be added to the short list, further discussions were held until consensus was reached.

##### Identifying What Needs to Change

Once the target behaviours had been selected, a ‘behavioural analysis’ was undertaken utilising the COM-B model of behaviour. This exercise is central to the BCW approach and involved the team drawing upon their experience and expertise and ethnography study findings to consider whether an individual’s capability, opportunity or motivation would need to be influenced for the target behaviours to occur.

#### Stage 2: Identifying Intervention Options

The next stage was to identify the most appropriate intervention functions to incorporate in the intervention that would have the best chance of influencing capability, opportunity or motivation; based on the behavioural analysis described above, available resources and contextual factors. The BCW offers the following suggestions of potential intervention functions: education, persuasion, incentivisation, coercion, training, restriction or environmental restructuring. To assist with decision-making around which intervention functions to include, the BCW suggests the use of the APEASE criteria (Michie et al. 2014) as a decision-making tool: affordability, practicability, effectiveness, acceptability, side effects and equity which the team used to structure group discussions.

#### Stage 3: Identifying Content and Implementation Options

The next stage was to decide on which behaviour change techniques to include (Michie et al. 2013). The BCW matches each potential intervention function selected in Stage 2 to a list of appropriate behaviour change techniques based on a consensus reached by experts in behaviour change (Michie et al. 2011). The intervention development team considered the evidence within the context of the children’s centre/local authority setting and again drew upon APEASE criteria and their own experience of HENRY and children’s centres to decide on the final behaviour change techniques to include.

Once the intervention function and behaviour change techniques had been selected, the most appropriate and realistic mode of delivery was agreed.

## Results

### Literature Review

The review identified five types of engagement interventions that had been tested to promote engagement with a public health programme: incentives, programme setting, manipulated promotional strategies, text message reminders and testimonials. Overall, none of the intervention types was consistently effective at promoting all stages of engagement, but monetary incentives were largely successful at promoting enrolment (Diaz and Perez 2009; Dumas et al. 2010; Heinrichs 2006; Hennrikus et al. 2002) and text message reminders were effective at promoting completion rates (Murray et al. 2015). This indicated that a multi-component intervention may be needed to enhance engagement at various stages.

## Stage 1

### Defining the Problem in Behavioural Terms

The results of the ethnography study are reported elsewhere (Burton et al. 2019), and therefore, only a brief summary is provided here to support describing the intervention development. The findings of the ethnography were consistent with what has previously been reported in the literature regarding participant level barriers to engagement with parenting programmes, e.g. programme acceptability, group dynamics and the personal attributes of the group facilitator (Beatty and King 2008; Friars and Mellor 2009; Gross et al. 2001; Owens et al. 2007; Pearson and Thurston 2006; Wheatley et al. 2003;). The study also revealed that engagement with HENRY was influenced by implementation factors that were present across multiple operational levels within the children’s centre/local authority context. In particular, a hierarchical spill-over affect was observed, whereby local authority ‘buy-in’ of HENRY cascaded down to children’s centre implementation of the programme which subsequently influenced how participants perceived and experienced the programme. A further finding of the ethnography study revealed that, although stakeholders acknowledged that some behaviours were likely to facilitate participant engagement with HENRY (e.g. HENRY training for all staff), practical barriers such as funding availability and capacity limited their ability to adopt them. Therefore, the problem defined in behavioural terms as to why centres struggled to recruit and retain participants on the HENRY programmes was that children’s centre stakeholders (commissioners, managers and centre staff) did not (or were not able to) adopt behaviours that were likely to promote participant engagement.

### Selecting the Target Behaviour

The shortlisting exercise resulted in a list of target behaviours proposed to promote engagement with HENRY that were to be performed by commissioners, managers, staff, HENRY facilitators and HENRY parents (parents that had previously attended HENRY) (Table 1). This included the delivery of ‘taster’ sessions prior to each delivered programme so that parents would gain a full understanding of what the programme entailed prior to enrolling, and the provision of HENRY training for all staff working in the centres so that they could provide an accurate representation of the programme when approaching parents to attend.

**Table 1:** Target behaviours for promoting parent engagement with HENRY

Parent engagement strategies	To be performed by	Rationale	Informed by
1. Hold ‘taster’ sessions prior to each HENRY programme (an opportunity for parents to meet facilitator and learn what the programme entails by receiving a ‘taster’ of a typical session	Children’s centre manager	Potential participants are more likely to engage if they have a greater understanding of what the programme entails	Experience of HENRY personnel, ethnography study finding (observation) and the literature, e.g. Gilbert et al. 2017
2. Increase HENRY training provision for centre staff	Children’s centre manager with the support of local authority commissioners	Some children’s centre staff lack basic knowledge of the content of the HENRY programme and would benefit from training on the HENRY approach	Ethnography study (interviews and observation), experience of team members and the literature, e.g. Davis et al. 2012 and Blaine et al. 2017
3. Hold HENRY programmes regularly and plan far in advance	Children’s centre manager with the support of local authority commissioners	Some HENRY programmes are planned at short notice which hinders recruitment efforts	Ethnography study (informal conversations) and experience of intervention development team
4. Promote HENRY widely in centres using a range of methods	Children’s centre manager	There is a general lack of awareness of HENRY among visiting parents	Ethnography study (observations, informal conversations and parent focus groups)
5. Allow a mix of referred and self-referred parents to enrol	Children’s centre manager with the support of local authority commissioners	Delivering programmes to a mix of parents (referred and self-referred) reduces barriers associated with stigma and improves group dynamics	Ethnography study (interviews and observations) and the literature (Bloomquist et al. 2013)
6. Adopt a whole centre approach to HENRY; whereby HENRY is well supported in the centre and HENRY principles are adopted in other programmes	Children’s centre manager	Adopting a whole centre approach to HENRY implementation achieves better outcomes for engagement	Ethnography study (observations and informal conversations) and experience of the intervention development team
7. Promote HENRY accurately to dispel myths and negative perceptions	Children’s centre staff	Misconceptions around what HENRY entails may deter people from engaging	Ethnography study (interviews, observations, focus group and informal interviews)
8. Ensure parents feel comfortable when attending the session	HENRY facilitators	The skills of facilitators are known to influence engagement	Ethnography study (observation, focus groups and interviews) and the literature, e.g. Owens et al., 2003 and Beatty et al., 2012
9. Follow up on all parents that miss a session to encourage continued attendance	HENRY facilitators	Participants feel valued if they are followed up after missing a session	Ethnography study (focus groups) and experience of the intervention development team
10. Encourage friends and family to engage with HENRY	Previous HENRY participants	Parent are more likely to attend a programme if they know someone that has attended before	Ethnography study (interviews and focus groups) and the literature, e.g. Gross et al., 2001and Friars et al., 2009

### Identifying What Needs to Change

The COM-B behavioural analysis determined the direction of the intervention at each level (Table 2). For example, it was agreed that centre managers were *capable* of adopting the target behaviours proposed to promote engagement with HENRY, but in order to adopt them, they would need to have the relevant support (*social opportunity*) from local authority commissioners, e.g. financial support. In addition, managers would need to be *motivated* to adopt them. Therefore, in order for the target behaviours to occur at the manager level, the intervention would need to influence *social opportunity* and *motivation.*

**Table 2:** Summary of behavioural analysis to identify which components of the COM-B model would need to be influenced in the participant engagement Intervention

Intervention level	Target behaviours	The COM-B construct that need to be influenced for target behaviours to occur		Would need to be influenced for behaviour change to occur	Potential intervention function suggested by BCW
Commissioner	Support managers to adopt target behaviours	Capability (psychological)	Commissioners need greater understanding of HENRY outcomes to facilitate decision making around level of support they are willing to provide	✓	Education, training or enablement
		Opportunity (physical)	Strict budgets exist around how much money can be invested into participant engagement efforts.	Maybe	Training, restriction, environmental restructuring, enablement
		Motivation (reflective)	Motivation of commissioners needs to be increased before additional resources are invested into participant engagement efforts	✓	Education, persuasion, incentivisation, coercion
Managers	1.Hold taster sessions prior to each HENRY programme Increase HENRY training provision for centre staff Hold HENRY programmes regularly and plan far in advance Promote HENRY widely within Centre using a range of methods Allow a mix of referred and self-referred parents to enrol Adopt a whole centre approach to HENRY	Capability (psychological)	Managers are already capable of performing the behaviours	*X*	N/A
		Opportunity (social)	Managers need support from commissioners before investing greater resources into parent engagement efforts	✓	Restriction, environmental restructuring, modelling, enablement
		Motivation (reflective)	Prior to investing greater resources into HENRY, manager’s motivation would need to be increased due to restricted budgets and staff capacity	✓	Education, persuasion, incentivisation, coercion
Children’s centre staff	Promote HENRY accurately to dispel myths about HENRY being a healthy eating programme	Capability (psychological)	Children’s centre staff often do not have the relevant capacity to perform the behaviours due to a lack of training	✓	Education, training or enablement
		Opportunity (Social)	Staff would require adequate social support from managers and team members to perform the behaviours, along with physical resources to assist with promoting the programme	✓	Restriction, environmental restructuring, modelling, enablement
		Motivation (reflective)	The motivation of some staff members would need to be increased in order for them to learn and implement new practices	✓	Education, persuasion, incentivisation, coercion
HENRY facilitators	1.Ensure parents feel comfortable when attending the session Follow up on all parents that miss a session to encourage continued attendance	Capability (psychological)	Some facilitators may lack the relevant capability to perform the behaviours, e.g. due to lack of experience	✓	Education, training or enablement
		Opportunity (physical)	A lack of time may present barriers to facilitators’ performing the behaviours	✓	Training, restriction, environmental restructuring or enablement
		Motivation (reflective)	The motivation of some facilitators could be increased to in order for them to invest additional time to HENRY planning	✓	Education, persuasion, incentivisation, coercion
Previous participants of HENRY	Encourage friends and family (peers) to engage with HENRY	Capability (psychological)	Previous participants of HENRY have the relevant capacity to be able to recruit their peers	*X*	N/A
		Opportunity (physical)	The relevant physical resources would need to be provided in order for previous participants of HENRY to recruit their peers. In addition, social support from centre managers would also need to be influenced so that parents feel comfortable that their peers would be eligible and welcome to attend	✓	Training, restriction, environmental restructuring or enablement
		Motivation (reflective)	Previous participants of HENRY that have enjoyed the programme would be motivated to recruit their peers. However, some may worry about causing offence, by inferring that the family/child needed to attend an obesity prevention programme	✓	Education, persuasion, incentivisation, coercion

## Stage 2: Identifying Intervention Options

The team agreed that the intervention would *educate* commissioners on why HENRY was beneficial to families in their community to increase their buy-in with the programme. It was also agreed that the intervention would *educate* them on the benefits of adopting the target behaviours in terms of promoting cost-effectiveness and programme reach. The intervention also aimed to *enable* commissioners to provide support to managers by providing them with data on the outcomes achieved by families that attend (e.g. changes to eating habits) so that they could make informed decisions about how much resource should be invested into engagement efforts. Gaining support from commissioners was proposed to *enable* managers to adopt the target behaviours. The intervention also aimed to motivate managers to adopt the target behaviours by *persuading* them on why it would be beneficial to do so. Similarly, gaining appropriate buy-in from managers was proposed to *enable* staff members to promote HENRY accurately, e.g. through means of training provision. The intervention also aimed to *persuade* staff members to promote HENRY accurately by encouraging managers to share information with them on how HENRY benefits families that attend. At the facilitator lever, it was agreed that facilitators would be *trained* on how to adopt the target behaviours, along with *persuading* them to do so by providing information on the expected benefits. Parents that had attended a HENRY programme would be *educated* on why it would be beneficial for them to recruit their peers, along with them being *enabled* to do so by providing them with any resources or support they might need.

## Stage 3: Identifying Content and Implementation Options

Behaviour change techniques selected to carry out each intervention function are described in Table 3 along with the associated intervention component.

**Table 3:** Participant engagement intervention selected intervention functions and behaviour change techniques linked to intervention component

Intervention level	Intervention function	Behaviour change technique	Detail	Intervention component
Commissioner	Enablement	12.5 Adding objects to the environment	Provide data on how HENRY benefits families that attend to guide decision making around HENRY investment	Commissioner report
	Persuade	5.6 Information on social consequences	Provide information on the benefits of promoting engagement with HENRY, how HENRY aligns with national public health targets and the benefits to families that attend	Commissioner leaflet and report
Managers	Persuasion	5.6 Information about social and environmental consequences	Provide information on the benefits of adopting target behaviours along with information on how HENRY benefits families that attend	Manager information day and dashboard report
		2.7 Feedback on outcome of behaviour	Provide feedback on how many parents enrolled and attended the HENRY programme	Dashboard report
	Enable	1.4 Action planning	Encourage managers to plan how they will implement target behaviours	Manager information day
		1.3 Goal setting	Encourage managers to set a goal for how often/to what degree they will implement target behaviours	Manager information day
Children’s Centre staff	Enable	12.5 Adding objects to the environment	Provide resources to enable children’s centre staff to promote HENRY accurately	Promotional material
	Persuasion	5.6 Information about social and environmental consequences	Provide information on how HENY benefits families that attend	Dashboard report
Intervention level	Intervention function	Behaviour change technique	Detail	Intervention component
HENRY facilitators	Training	4.1 Instruction on how to perform the behaviour	Advise HENRY facilitators on how to perform target behaviours	Facilitator refresher training
		6.1 Demonstration of the behaviour	Demonstrate how to perform target behaviours	Facilitator refresher training
	Persuasion	5.6 Information about social and environmental consequences	Provide information on the benefits of adopting the target behaviours	Facilitator refresher training
Parents that have attended HENRY	Enablement	12.5 Adding objects to the environment	Provide resources to enable HENRY parents to recruit their peers	Promotional material
	Education	5.6 Information on social consequences	Provide information on the benefits of adopting peers	Information provided by HENRY facilitator

### Participant Engagement Intervention Components

The HENRY participant engagement intervention comprises six components: commissioner outcome report, commissioner overview leaflet, manager dashboard report, manager information workshops, HENRY facilitator refresher training and revised promotional material. As mentioned, specific details on behaviour change techniques delivered within each intervention component are provided in Table 3.

#### Commissioner Report

Existing processes at HENRY central office included the provision of outcome data to commissioners HENRY prior to the intervention. However, during the ethnography study, it was revealed that these data were not received often enough and at the appropriate time points to assist with decision-making around levels of investment. Therefore as part of the information, reporting procedures were tightened so that commissioners would receive an outcome report quickly at the end of each programme delivery period (usually delivered in line with school periods, i.e. four monthly). Outcomes included in the report are enrolment and attendance, participant feedback and behaviour change outcomes from the start to the end of the programme (e.g. changes to family eating habits).

#### Commissioner Overview Leaflet

The commissioner overview leaflet was designed to increase local authority buy-in with HENRY and the participant engagement intervention by providing commissioners with information on how HENRY aligns with national public health targets and the proposed benefits of managers adopting the target behaviours. The leaflet is circulated to commissioners that deliver HENRY programmes prior to the start of the intervention delivery period to gain support for intervention activities.

#### Dashboard Report

The dashboard report is a one-page report designed to persuade managers to adopt the target behaviours. The report is sent to all managers that deliver HENRY within their children's centre at the start of the intervention and after each delivered programme thereafter. The report provides feedback to managers on parent engagement outcomes achieved for the previous HENRY programme and summarise behaviour change outcomes achieved by the parent e.g. changes in parent and child fruit and vegetable intake as a result of attending. Managers are also encouraged to share the information provided in the report with centre staff so that they can also made aware of the benefits to families as a result of attending HENRY.

#### Manager Information Workshops

The manager information workshop was designed to be attended by all managers that deliver HENRY programmes within their centre. The 1-day workshop is delivered at the start of the intervention. During the workshops, managers are briefed on the aims of the HENRY participant engagement intervention and proposed logic model. Group discussions and activities also take place around the proposed benefits of adopting the target behaviours, goal setting and action planning on how the behaviours could be implemented within their setting. Managers also have the opportunity to discuss anticipated barriers to performing the behaviours and share knowledge on how these may be overcome.

#### Facilitator Refresher Training

Facilitator refresher training was deigned to be offered to all HENRY facilitators within a local authority that delivered HENRY programmes. The interactive training takes place over one full day where facilitators are briefed on the aims of the HENRY participant engagement information and the proposed benefits of adopting the target behaviours. Training and demonstrations on how to adopt the target behaviours are also provided. During the workshop, facilitators are also instructed to introduce ‘peer’ recruitment to parents that attend HENRY to encourage them to recruit their friends and family.

#### Revised Promotional Material

Existing HENRY promotional material was revised to more accurately portray what the HENRY programme entails. Included in this was this was a change to the tagline displayed on all promotional material from ‘Health, Exercise and Nutrition for the Really Young’ to ‘Healthy Family, Happy Home’ to better depict the holistic nature of the programme. The promotional material was designed to be displayed in all children’s centres delivering HENRY to attract potential participants. In addition, the promotional material also aimed to support children’s centre staff to accurately portray the programme and provide a resource for HENRY parents to be able to recruit their peers to the programme.

A logic model was developed by the intervention development team to outline how the participant engagement intervention proposed to promote engagement with HENRY (Online Resource 1). In brief, adoption of the target behaviours by commissioners, managers, staff, HENRY facilitators and HENRY parents proposed to increase support of parent engagement efforts; increase awareness and understanding of the programme among potential participants and centre staff; normalise the HENRY programme within the children’s centre and community, reducing stigma and negative perceptions; and optimise the participant experience to promote engagement during HENRY sessions, thus achieving greater reach and impact of the programme along with increased sustainability and cost-effectiveness.

## Discussion

This paper describes the development of a theory-based participant engagement intervention aimed at supporting local authorities to promote engagement with a community delivered obesity prevention programme. Participant engagement with preventative public health programmes is central to achieving meaningful impact, yet there is a lack of studies rigorously evaluating the effect of strategies aimed at promoting engagement, and from those that have, few found a positive effect.

The majority of reported participant engagement interventions in the literature comprise of single strategies directed only at anticipated beneficiaries which are largely ineffective. Moreover, although reported strategies are mostly theoretically based, they are often not tailored to address particular barriers identified within a programme’s context. Participant engagement is likely influenced by multiple contextual factors such as organisational strategies, local implementation practices, intervention characteristics and the characteristics of individuals involved in a programme’s delivery (Rogers 1962, Damschroder et al. 2009, Burton et al. 2019). Thus, in theory, interventions aimed at multiple organisational levels have greater potential for promoting participant engagement with public health programmes. This participant engagement intervention addresses the multiple levels of influence that hinder effective programme implementation of HENRY. To our knowledge, this is the first study that has adopted this approach with the primary aim of optimising participant engagement with a public health programme.

The BCW provided a useful guide to develop this participant engagement intervention, offering valuable decision-making tools such as APEASE criteria. However, the focus on individual behaviour change directed by the COM-B model of behaviour was sometimes difficult to apply to a whole setting approach where hierarchical structures influence whether behaviour change is possible. In future, combining the BCW with another theoretical model may be beneficial. For example, Band et al. (2017) successfully utilised both the COM-B model of behaviour and Normalisation Process Theory (May et al. 2009) to develop an intervention which considered both individual level and organisation level factors in its design that was most relevant to the user population and setting.

The participant engagement intervention was designed using a rigorous and transparent process. Consulting with a parent advisory group was invaluable in learning how the wider impact of the intervention could ultimately influence participant engagement with HENRY. Incorporating an ethnography study also provided a thorough understanding of the setting in which HENRY is delivered which enabled a tailored intervention to be developed that addresses specific implementation barriers to participant engagement. The methods and insight gained through the development of the participant engagement intervention could be applied to other public health programmes delivered within a community setting. A limitation of the intervention development process was that stakeholders from the children’s centres were not involved in decision-making about the final intervention functions and components. However, they were important in identifying where intervention was needed through their ongoing involvement in the ethnographical research. Implementation of the participant engagement intervention did not include a piloting phase. Ideally, any intervention should be piloted prior to full implementation, but due to timeline and resources, this was not done here which is a recognised limitation.

The participant engagement intervention is currently being tested in a multi-site, cluster randomised controlled trial (Bryant et al. 2017); the results of which will be reported elsewhere when available. A comprehensive process evaluation will also report on the implementation of the intervention and explore the change mechanisms. Throughout the development of the participant engagement intervention, the development team have been mindful of the severe upheaval that has occurred throughout local authorities and children’s centre services in England in recent years which have led to substantial re-structuring and job losses (Sammons et al. 2015). The influence of these contextual factors on the implementation of programmes such as HENRY is yet unknown.

## Conclusions

This paper describes an example of one of the first attempts to develop a multi-level participant engagement intervention designed to promote participant engagement with an obesity prevention programme, using HENRY as an example. Highlighted within the development process was the importance of identifying the barriers and levels within the implementation setting that promoted or hindered participant engagement. The use of the BCW framework served as a useful guide to consider which behavioural components needed to be influenced for behaviour change to occur before providing a transparent and systematic decision-making tool. Given the challenges to implementing public health programmes with sufficient reach, the process used to develop the participant engagement optimisation intervention serves as an example of how programmes that are already widely commissioned and have the potential to improve the health of the population could be optimised to enable greater impact.

## Data Availability

Not applicable.
